# Dietary intervention, biomarkers of brain pathology and neurodegeneration, and cognition: The MIND Trial Study

**DOI:** 10.1002/alz.087564

**Published:** 2025-01-09

**Authors:** Klodian Dhana, Kumar B Rajan, Neelum T. Aggarwal, Konstantions Arfanakis, Vince J Carey, Frank Sacks, Lisa L. Barnes

**Affiliations:** ^1^ Rush University Medical Center, Chicago, IL USA; ^2^ Rush Institute for Healthy Aging, Chicago, IL USA; ^3^ Rush Alzheimer’s Disease Center, Chicago, IL USA; ^4^ Illinois Institute of Technology, Chicago, IL USA; ^5^ Harvard Medical School, Boston, MA, Boston, MA USA; ^6^ Harvard T. H. Chan School of Public Health, Boston, MA USA

## Abstract

**Background:**

Plasma levels of phosphorylated tau (pTau‐181), neurofilament light chain (NfL), and glial fibrillary acidic protein (GFAP) as biomarkers of brain pathology and neurodegeneration have been associated with cognition and Alzheimer’s dementia. Whether a dietary intervention modifies these relationships by potentially promoting cognitive reserve is unknown. We conducted a posthoc analysis of the MIND trial to investigate whether dietary intervention modifies the association of longitudinal changes in pTau‐181, NfL, and GFAP with cognition during 3 years of follow‐up.

**Method:**

MIND trial was a 3‐year, two‐site, randomized controlled clinical trial comparing the MIND diet with a usual diet on cognitive changes among 604 older participants (65 to 85 years) with a family history of dementia but without cognitive impairment, overweight, and with suboptimal diets at enrollment. pTau‐181, NfL, and GFAP were measured in plasma from blood collected after an overnight fast. Relative change in biomarkers (i.e., %change) from baseline to Year 3 were computed [(year 3 ‐ baseline)/baseline)*100]. Global cognition was derived from a standardized 12‐test cognitive battery. We included 508 individuals with biomarkers and cognitive assessments at the baseline and year 3.

**Result:**

Changes in pTau‐181 and NfL were associated with global cognition. One SD increase in %change of pTau‐181 was associated with 0.049 SD units lower in cognition at year 3 ($∖beta$ ‐0.049; 95%CI ‐0.081 to ‐0.016). For NfL, cognition was 0.044 SD units lower at year 3 (β ‐0.044; 95%CI 0.076 to ‐0.012). The strength of these associations differed by the dietary arm (p for interaction = 0.063), such that effects were attenuated in the MIND diet arm and more robust in the control arm. Specifically, pTau‐181 in the MIND diet group was ‐0.027 (p = 0.264), and in the control group, ‐0.076 (p = 0.001). For NfL, these estimates were ‐0.022 (p = 0.263) and ‐0.087 (p = 0.002) in the MIND and control diet arms, respectively.

**Conclusion:**

Levels of biomarkers reflecting neurodegeneration increase with age, and we showed that the greater increase was associated with lower cognitive scores. Among individuals with greater changes in biomarkers, MIND diet intervention attenuated the association with cognition, suggesting that the MIND diet may promote cognitive reserve in older adults.

